# FIVE BIODEMOGRAPHIC PRINCIPLES OF AGING THAT EMERGED FROM RESEARCH ON THE MEDITERRANEAN FRUIT FLY

**DOI:** 10.1093/geroni/igad104.0199

**Published:** 2023-12-21

**Authors:** James Carey

**Affiliations:** University of California, Davis, Davis, California, United States

## Abstract

Because of its large size, visually-distinct sex differences, abundance in the wild and ease of rearing at both industrial and individual levels, the Mediterranean fruit fly (medfly) is an ideal model organism for conducting research on the demography of aging. I will present details on and implications for what I consider to be the five most interesting and important aging-related discoveries from the NIA-funded research my colleagues and I conducted over the past three decades using the medfly model. These discoveries include (1)Deceleration of mortality at advanced ages; (2)Equivocality of the gender gap due to context-specific sex mortality relationships; (3)Supine behavior as a biomarker of both morbidity onset and time-to-death; (4)A dietary disconnect between maximizing longevity vs maximizing lifetime reproduction; and (5)the life table population identity in which the fraction age x in a stationary population equals the fraction with x years to live. I will end with brief comments about the use of non-conventional model organisms for research on aging in both the laboratory and the field.

